# A Luminacin D Analog HL142 Inhibits Ovarian Tumor Growth and Metastasis by Reversing EMT and Attenuating the TGFβ and FAK Pathways

**DOI:** 10.7150/jca.61066

**Published:** 2021-07-25

**Authors:** Baojin Wang, Hanxuan Li, Xinxin Zhao, Wenjing Zhang, Guannan Zhao, Zhongzhi Wu, Ruitao Zhang, Peixin Dong, Hidemichi Watari, Gabor Tigyi, Wei Li, Junming Yue

**Affiliations:** 1 Department of Gynecology and Obstetrics, Third Affiliated Hospital, Zhengzhou University, Zhengzhou 450052, China; 2 Department of Pathology and Laboratory Medicine, College of Medicine, the University of Tennessee Health Science Center, Memphis, TN, 38163, USA; 3 Center for Cancer Research, College of Medicine, the University of Tennessee Health Science Center, Memphis, TN, 38163, USA; 4 Department of Pharmaceutical Sciences, College of Pharmacy, the University of Tennessee Health Science Center, Memphis, TN, 38163, USA; 5 Department of Genetics, Genomics & Informatics, College of Medicine, the University of Tennessee Health Science Center, Memphis, TN, 38163, USA; 6 Department of Gynecology and Obstetrics, First Affiliated Hospital, Zhengzhou University, Zhengzhou 450052, China; 7 Department of Gynecology, Hokkaido University School of Medicine, Hokkaido University, Sapporo, Japan; 8 Department of Physiology, College of Medicine, the University of Tennessee Health Science Center, Memphis, TN, 38163, USA

**Keywords:** Luminacin D analog, HL142, EMT, ovarian tumor, metastasis, ASAP1, FAK

## Abstract

Epithelial to mesenchymal transition (EMT) is known to contribute to tumor metastasis and chemoresistance. Reversing EMT using small molecule inhibitors to target EMT associated gene expression represents an effective strategy for cancer treatment. The purpose of this study is to test whether a new luminacin D analog HL142 reverses EMT in ovarian cancer (OC) and has the therapeutic potential for OC. We chemically synthesized HL142 and tested its functions in OC cells *in vitro* and its efficacy in inhibiting ovarian tumor growth and metastasis *in vivo* using orthotopic OC mouse models. We first demonstrate that ASAP1 is co-amplified and interacts with the focal adhesion kinase (FAK) protein in serous ovarian carcinoma. HL142 inhibits ASAP1 and its interaction protein FAK in highly invasive OVCAR8 and moderately invasive OVCAR3 cells. HL142 inhibits EMT phenotypic switch, accompanied by upregulating epithelial marker E-cadherin and cytokeratin-7 and downregulating mesenchymal markers vimentin, β-catenin, and snail2 in both cell lines. Functionally, HL142 inhibits proliferation, colony formation, migration, and invasion. HL142 also sensitizes cell responses to chemotherapy drug paclitaxel treatment and inhibits ovarian tumor growth and metastasis in orthotopic OC mouse models. We further show that HL142 attenuates the TGFβ and FAK pathways *in vitro* using OC cells and *in vivo* using orthotopic mouse models.

## Introduction

Epithelial to mesenchymal transition (EMT) is a biological process by which epithelial cells lose cell polarity and acquire invasive mesenchymal stem cell properties. Molecularly, the EMT phenotypic switch is accompanied by downregulation of epithelial cell markers including E-cadherin and cytokeratin, and upregulation of mesenchymal markers such as vimentin, fibronectin, snails, and N-cadherin 1, 2. EMT is regulated by complex molecular pathways and multiple signaling events including TGFβ, WNT, MAPK, FAK and JAK/Stat, Hedgehog, and Hippo-YAP/TAZ 3, 4. Accumulating experimental evidence indicates that EMT contributes to tumor metastasis, chemoresistance, and immunosuppression 1, 5, 6. EMT marker gene expression levels are associated with different ovarian cancer (OC) grades or stages and tumor heterogenicity^7^. EMT also contributes to OC metastasis and chemoresistance 7-12. Ovarian tumor metastasis undergoes an EMT/MET (mesenchymal to epithelial transition) conversion that primary ovarian tumor cells shed into peritoneum and form mesenchymal spheroids or aggregates (EMT) and then metastasize into distant organs where cells switch back to epithelial cells (MET), which allows cells to proliferate in metastasized organs 13. Multiple genes have been identified to promote EMT in OC such as Stat4, twist, CD24, and CD44, and targeting those gene expressions reverses EMT and has great potential for OC therapy 14-18.

Several approved drugs have been tested or repurposed to reverse EMT for cancer therapy. For example, denosumab (a NF-kB inhibitor), WP-1066 (a STAT3 inhibitor), and PEGPH20 (a HIF-1α inhibitor) are currently in clinical trials 19. A few other EMT targeting drugs are also in clinical trials 20. Some metabolic inhibitors have been shown to reverse EMT. For example, Rolipram, a selective inhibitor of PDE4 with antipsychotic effects, and Simvastatin, a competitive inhibitor of 3-hydroxy-3-methylglutaryl-coenzyme A reductase reverse EMT by inhibiting the TGFβ pathway 21.

ASAP1 (ADP ribosylation factor ARF GTPase-activating protein GAP containing SH3, ANK repeats, and PH domain) has been shown to interact with the pleckstrin homology (PH) or the canonical Src homology 3 (SH3) domains of interacting protein 22, 23. ASAP1 is rarely expressed in normal tissue but is highly expressed in tumors and is well-correlated with tumor metastasis in several cancer types 24-27. ASAP1 expression promotes tumor metastasis and chemoresistance by interacting with other oncogenic proteins. ASAP1 is located in the 8q24 genomic locus associated with tumor metastasis and recurrence 26, 27. ASAP1 interacts through its SH3 domain with the PH domain of PTK2 (FAK) 22. We and others have previously shown that ASAP1 promotes EMT in ovarian and other cancer types 28-30. Therefore, targeting ASAP1 has potential for OC therapy by reversing EMT.

Luminacin D is an antibiotic extracted from Streptomyces *sp* and has been identified as an inhibitor of the canonical SH3/proline binding and thus disrupts the SH3 domain-mediated intermolecular interaction in eukaryotic cells 31-35. A luminacin D analog, UCS15A, has been reported to inhibit breast cancer metastasis by disrupting the interaction of ASAP1 with its interacting partners cortactin and paxillin 36. In this study we report a finding that an analog of luminacin D, the compound 14 in a previous report 37, we denoted as HL142 has a similar structure with UCS15A. We synthesized HL142 and test its antitumor activities in OC and found that HL142 suppressed ovarian tumor growth and metastasis by inhibiting ASAP1, FAK and reversing EMT through attenuating the FAK and TGFβ pathways.

## Materials and Methods

### Cell culture

OC cell lines OVCAR-3 and OVCAR-8 were purchased from the National Cancer Institute and maintained in RPMI 1640 medium supplemented with 10% FBS (Hyclone, Logan, UT), 100 U/ml penicillin, and 100 μg/ml streptomycin (Invitrogen, Carlsbad, CA). Cells were authenticated by ATCC using Short Tandem Repeat (STR) analysis and tested negative for mycoplasma using luciferase assay (Lonza, Allendale, NJ). HL142 was synthesized as described previously and characterized as described earlier 37 .

### Cell migration assays

Cell migration was analyzed using modified Transwell chambers (BD Falcon™, San Jose, CA) inserted into 24-well culture plates. OVCAR-3 or OVCAR-8 cells (3×10^5^) were treated with 10 μM HL142 or vehicle for 24h and then suspended in 300 μL serum-free RPMI 1640 medium and added to the upper chamber. RPMI 1640 containing 10% FBS in medium as the chemical attractant was added in the bottom of the chamber and incubated for 24h. The upper chamber medium and non-migrated cells were removed, and migrated cells were fixed with methanol and stained with Crystal Violet. Images were taken at 10× magnification and migrated cells were counted in at least three randomly selected different fields using the ImageJ software.

### Cell invasion assays

OVCAR-3 or OVCAR-8 cells (3×10^5^) were treated with 10 μM HL142 or vehicle for 24h and then seeded in serum-free RPMI 1640 media onto inserts precoated with Matrigel (BD BioSciences, San Jose, CA). RPMI 1640 containing 10% FBS was added in the bottom chamber as chemoattractant and incubated for 48h and the invaded cells were stained and counted as described previously 38.

### Cell proliferation assay and cell viability

Cell proliferation was measured using an MTT kit purchased from ATCC (Manassas, VA) following the manufacturer's instructions. Briefly, OVCAR-3 or OVCAR-8 cells (3,000/well) were plated into 96-well plates and treated with different doses of HL142 or vehicle at different time points. Cell viability was measured with acridine orange (AO)/propidium (PI) solution. Live green and dead red cells were counted using a Luna-FL automatic cell counter.

### Cell clonogenic assay

OVCAR-3 or OVCAR-8 cells (300 cells/well) were plated into 6-well plates and treated with 10 μM HL142 or vehicle and cultured for two weeks and then stained with 0.1% Crystal Violet. Cell colonies were counted using ImageJ software, and then statistical analysis was performed from three different wells.

### Western blot (WB)

OC cells were collected in RIPA buffer (Thermo Scientific, Rockford, IL) containing 1% halt proteinase inhibitor cocktail (Thermo Scientific). Equal amounts of protein (40 μg/lane) were loaded onto 10% SDS-PAGE gels and transferred onto nitrocellulose membranes. The membranes were blocked with 5% nonfat milk for 1h and incubated with primary antibodies against ASAP 1 (1:1,000, Rockland; Atlanta, GA), E-cadherin, N-cadherin, vimentin, β-catenin, snail2, FAK, p-FAK, Cleaved-PARP, Cleaved-caspase3, SMAD2/3, pSMAD2 (1:1000, Cell Signaling Technology, Inc, Danvers, MA), Cytokeratin-7 (1:1,000, Abcam), and (GAPDH, 1:1,000, Sigma, St. Louis, MO) for 12h at 4°C. After washing three times with PBST for 5 min each, membranes were incubated with secondary antibody for 1h at room temperature. Band intensity in the Western blots was measured using ImageJ software.

### Immunoprecipitation assay (IP)

Cells were lysed with 25 mM Tris, 150 mM NaCl,1 mM EDTA, 1% NP-40 and 5% glycerol, pH 7.4 (lysis buffer). 100 µg of total protein was set aside as input, whereas 2 mg of total protein were pre-cleared with Control Agarose Resin from Pierce Classic IP Kit (Thermo Fisher) for 1h at 4℃, then incubated with either 10 µg of IgG or FAK or ASAP1 antibody (Santa Cruz) overnight at 4℃. The immune complex was then captured with Pierce Protein A/G Agarose beads for 1h at 4℃, washed four times with lysis buffer, one time with conditioning buffer, and eluted with elution buffer. The eluted fractions were analyzed by Western blot using ASAP1 or FAK antibodies.

### Mouse xenograft model

All animal experiments were conducted in accordance with the protocol approved by the Institutional Animal Care and Use Committee (IACUC) at the University of Tennessee Health Science Center. OVCAR-8 cells (1×10^6^) labeled with luciferase using a lentiviral vector expressing luciferase (pLenti-UBC-Luc2) were intrabursally injected into ten two-month-old immunocompromised NOD scid gamma (NSG) female mice. After one week of cell injection, mice were randomized into two groups. One group of mice was treated with HL142 (20 mg/kg body weight) and the other group of mice was treated with vehicle every other day. Tumor initiation and progression was monitored using a Xenogen animal imaging system weekly. After three weeks of treatment, all mice were sacrificed, tumors were weighed and collected for histology and Western blot to detect the expression of ASAP1, FAK, and EMT markers.

### Genetic alteration analysis of OC in TCGA database

To examine the genetic alteration of PTK2 and ASAP1, we examined genetic profiles of PTK2 and ASAP1 from two different datasets including the Firehose Legacy and PanCancer Atlas of ovarian cancer in TCGA database using the cBioPortal program 39.

### Statistical analysis

All data were analyzed using GraphPad Prism 7. Significant differences between at least two independent experiments were determined by one-way ANOVA and Student's *t*-test, and the data were expressed as mean ± SD. *P*<0.05 was considered as statistically significant.

## Results

### ASAP1 is co-amplified and interacts with FAK in ovarian carcinoma

**Figure 1:**
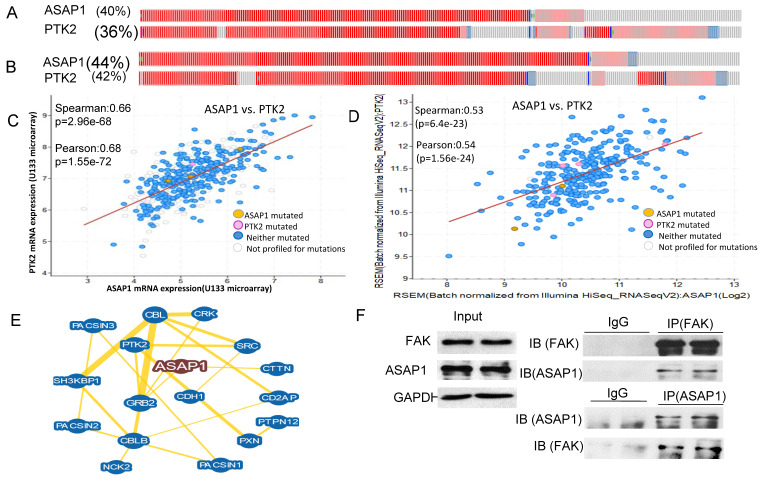
** ASAP1 is co-expressed with FAK in ovarian serous carcinoma. A, B.** ASAP1 and FAK copy numbers are co-amplified or upregulated with FAK in firehose legacy and Pan Cancer Atlas datasets of ovarian serous carcinoma, respectively. **C, D.** ASAP1 is co-expressed with FAK in in firehose legacy and Pan Cancer Atlas datasets of ovarian serous carcinoma, respectively. **E.** ASAP1 interacts with FAK, E-cadherin, Src, and other associated genes analyzed using the BioGRID database. **F.** The interaction of ASAP1 with FAK was validated in OVCAR8 cells by performing IP. ASAP1 and FAK antibodies were used to immunoprecipitate protein complex and then blotted with FAK and ASAP1 antibodies, respectively.

We showed previously that ASAP1 was highly expressed in ovarian carcinoma and was associated with poor patient survival 29. To understand how ASAP1 interacts with other associated proteins, we analyzed TCGA database on genetic alteration from two different OC datasets. In the firehose legacy dataset**,** 40% of 606 patients displayed ASAP1 upregulation or amplification and 36% of them showed FAK (PTK2) upregulation or amplification (Fig. 1A). In Pan Cancer Atlas, 44% of 585 patients exhibited ASAP1 upregulation or amplification and 40% of them had FAK upregulation or amplification in a subgroup of patients (Fig. 1B).

To understand whether ASAP1 upregulation and FAK amplification are correlated in these patients, we further analyzed the co-expression of ASAP1 and FAK in both datasets based on mRNA array or RNA-seq data and found that ASAP1 expression was well-correlated with FAK expression in both datasets (Fig. 1C, D). We also analyzed ASAP1 interaction using BioGRID database and found that ASAP1 interacts with FAK, E-cadherin (CDH1) and Src (Fig. 1E). To validate our hypothesis of the interaction between ASAP1 and FAK, we performed IP in OVCAR8 cells using ASAP1 or FAK antibodies and found that ASAP1 indeed physically interacts with FAK in OC cells (Fig. 1F). Our data indicated that ASAP1 is upregulated or amplified and interacts with FAK in OC.

### HL142 inhibits ASAP1 and EMT in OC cells

**Figure 2:**
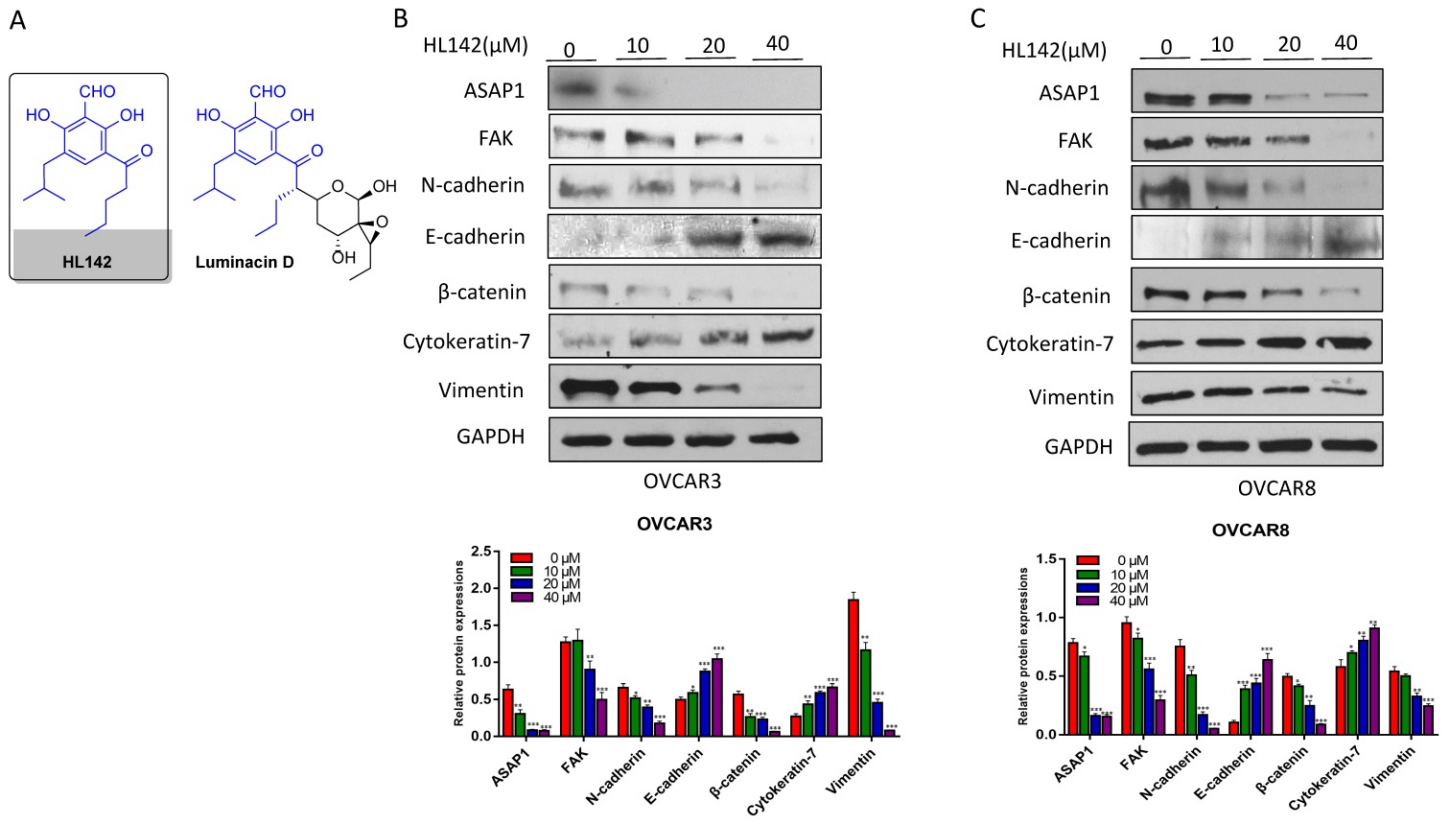
** HL142 inhibits EMT in OC cells. A.** Structure of HL142 and its analog luminacin D. **B, C.** WB and densitometry analysis of ASAP1 and EMT marker expression in OVCAR3 (A) and OVCAR8 cells (B) following different doses of HL142 treatment for 48h.

Based on the correlation of ASAP1 with FAK in ovarian carcinoma and oncogenic properties, it is an attractive approach to target both ASAP1 and FAK for OC therapy. HL142 is a synthetic analog of luminacin D (Fig. 2A), but its function has not yet been reported. Based on our previous finding that ASAP1 expression promotes EMT 29, we screened a collection of compounds in our lab, including HL142, for their potential efficacy to inhibit ASAP1 expression in OC cells. Serendipitously, we found that HL142 was active when we treated both OVCAR3 and OVCAR8 cells with different doses of HL142 for 48h. The ASAP1, FAK, and mesenchymal markers N-cadherin, β-catenin, vimentin and snail2 were reduced whereas the epithelial markers E-cadherin and cytokeratin-7 were increased in a dose dependent manner following HL142 treatment in both cell lines (Fig. 2B). These results indicate that HL142 inhibited ASAP1, FAK expression, and EMT in OC cells.

### HL142 inhibits proliferation, colony formation, migration, and invasion in OC cells

**Figure 3:**
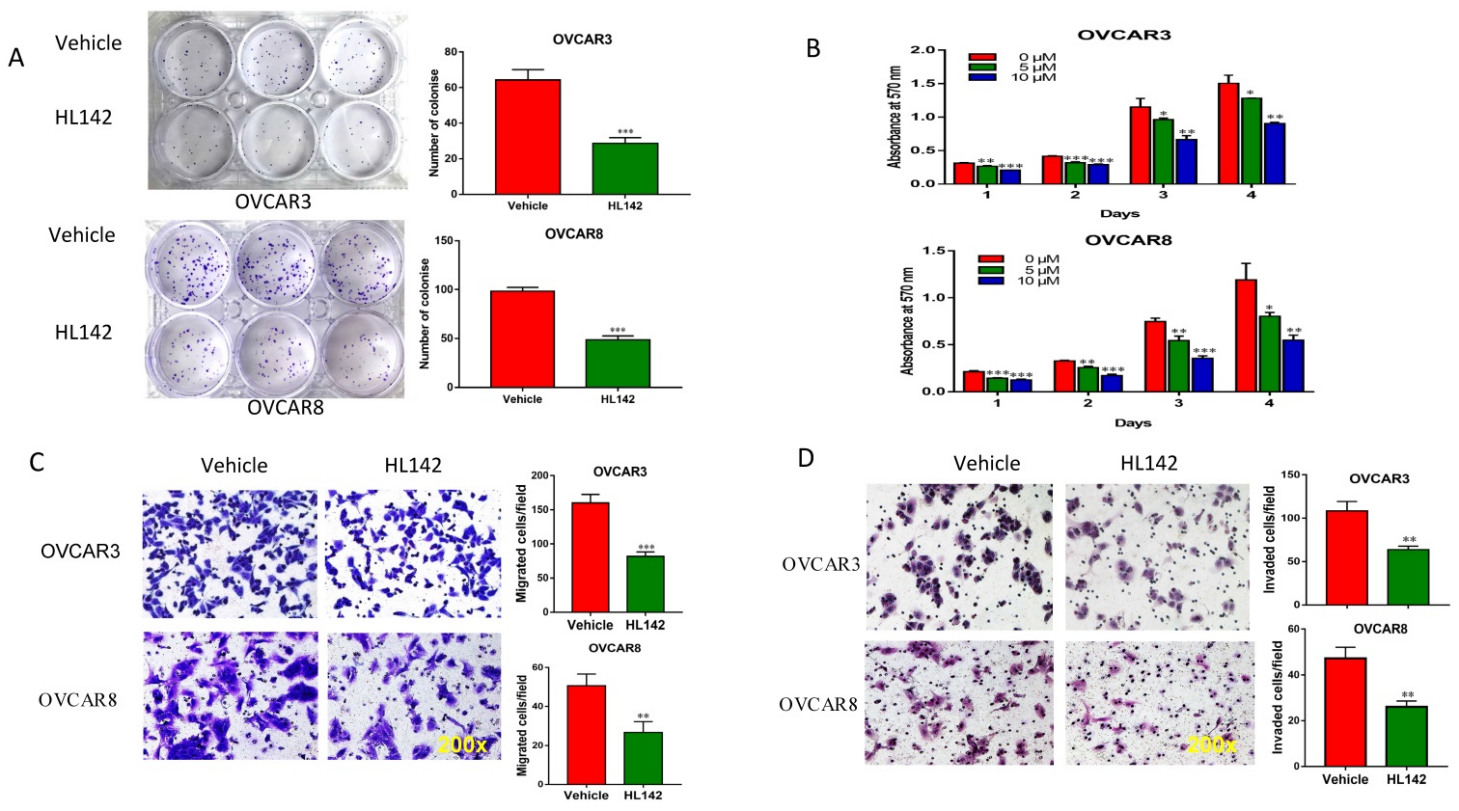
** HL142 inhibits proliferation, colony formation, migration and invasion in OC cells. A.** Cell colonies in OVCAR3 and OVCAR8 cells following 10 µM HL142-treatment for two weeks and stained with Crystal Violet. **B.** MTT analysis of cell proliferation in OVCAR3 and OVCAR8 cells treated with different doses of HL142 at different time points. **C.** Transwell cell migration assay of OVCAR3 and OVCAR8 cells treated with 10μM HL142 for 24h and migrated cells were stained with Crystal Violet. **D.** Matrigel-coated transwell invasion assay of OVCAR3 and OVCAR8 cells treated with 10μM HL142 for 48h and stained with H&E (***p<0.001, **p<0.01, *p<0.05).

To functionally evaluate whether HL142 inhibits OC cell growth, we performed cell clonogenic assays by treating OVCAR3 and OVCAR8 cells with 10 μM HL142 for two weeks and found that HL142 significantly inhibited colony formation in both cell lines (Fig. 3A). We also examined cell viability by performing AO/PI staining and found that HL142 did not affect cell viability in the 10 to 40 µM doses, but inhibited cell proliferation (Fig. S1, S2). We also examined cell proliferation by performing MTT assay following treatment of both OVCAR3 and OVCAR8 cells with different doses at different time points. Cell proliferation was significantly inhibited by HL142 compared with vehicle treated cells (Fig. 3B). We further examined the effect of HL142 on cell migration and invasion in both OVCAR3 and OVCAR8 cells. As shown in Fig. 3C and 3D, cell migration and invasion were significantly inhibited by 10 μM HL142 compared to the vehicle treated OVCAR3 and OVCAR8 cells. Our data indicated that functionally HL142 inhibits OC cell proliferation, migration, and invasion.

### HL142 induces cell apoptosis and enhances the efficacy of chemotherapy drugs in OC cells

**Figure 4:**
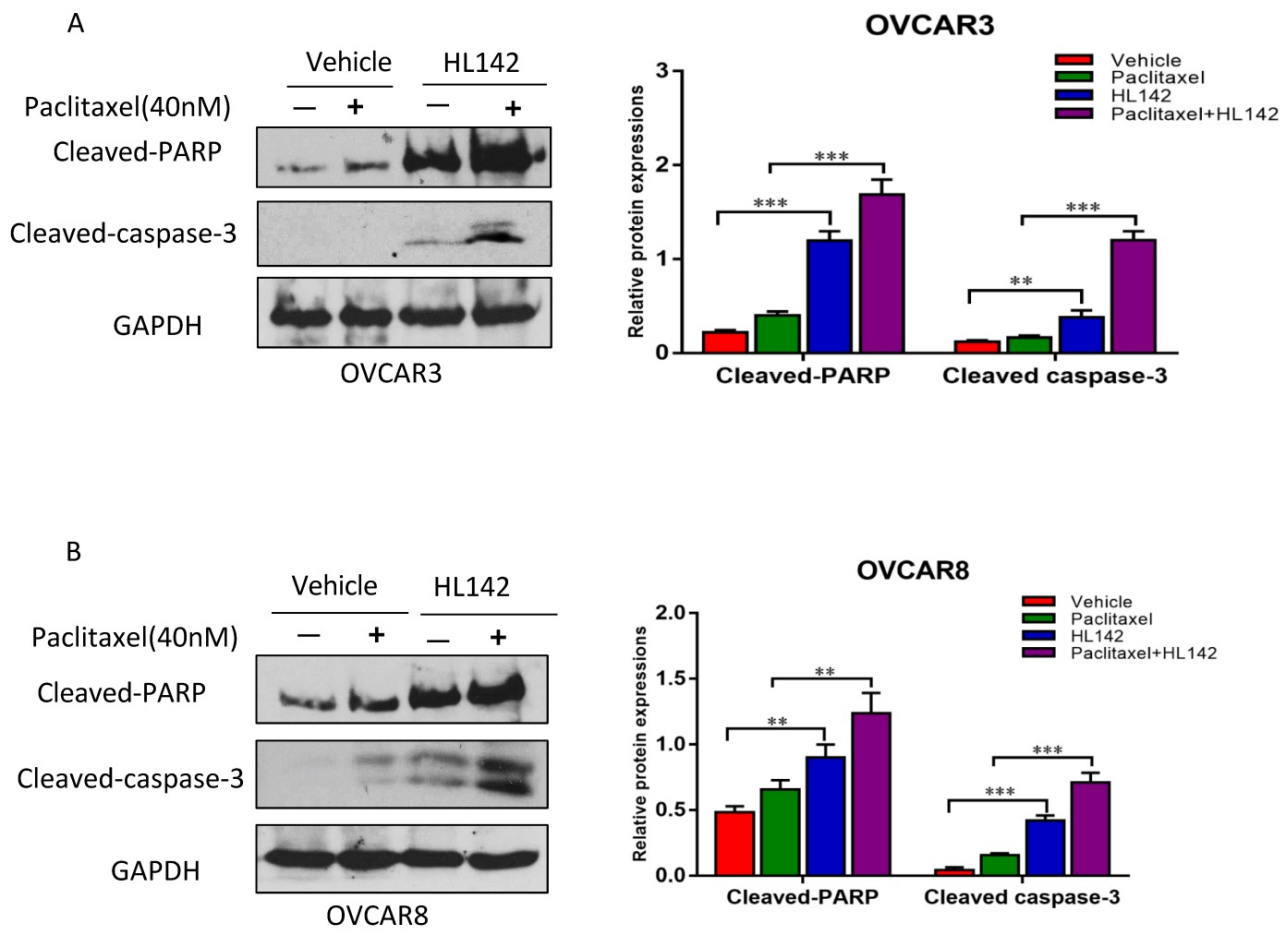
** HL142 promotes apoptosis induced by paclitaxel in OC cells. A, B.** WB and densitometry analysis of cell apoptosis by examining cleaved-PARP and -caspase3 in OVCAR3 and OVCAR8 cells treated with 10 µM HL142 or 40nM paclitaxel alone or both together (***p<0.001, **p<0.01).

We previously showed that overexpression of ASAP1 promotes chemoresistance in OC cells 29, and HL142 inhibited the expression of ASAP1 and cell proliferation. Thus, we tested whether HL142 induced cell apoptosis in OC cells. We treated OVCAR3 and OVCAR8 cells with 10 μM HL142, 40 nM paclitaxel, and their combination for 48h. After that, cell apoptosis was examined by measuring cleaved-PARP and cleaved-caspase3 using Western blotting. As shown in Fig. 4A and B, HL142 significantly induced cleaved PARP and cleaved caspase3. Our data indicated that HL142 sensitizes cell responses to paclitaxel treatment in both cell lines.

### HL142 attenuates the TGFβ and FAK pathways in OC cells

**Figure 5:**
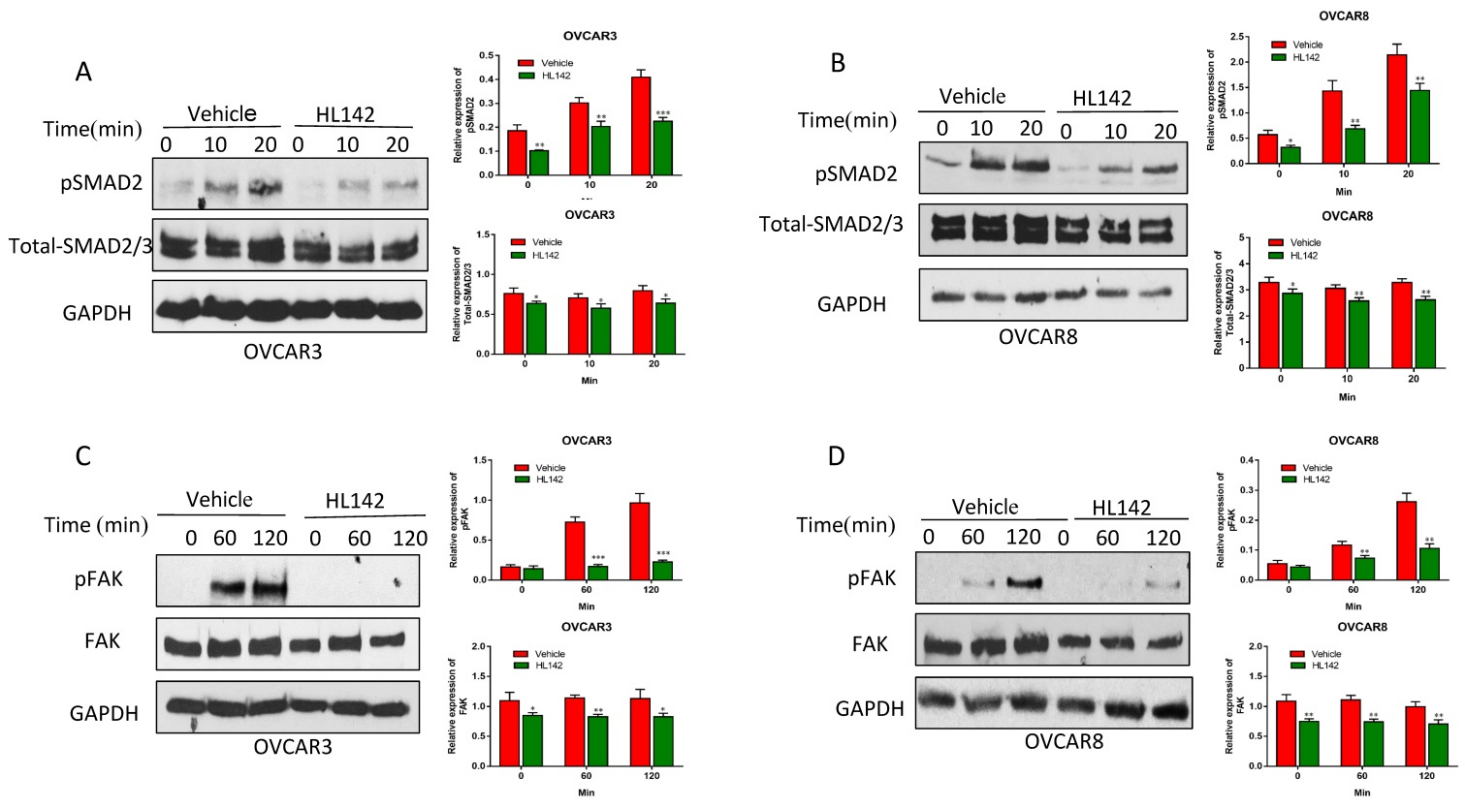
** HL142 attenuates the TGFβ/FAK pathway in OC cells. A, B.** WB and densitometry analysis of pSMAD2 and total SMAD2 expression in OVCAR3 and OVCAR8 cells treated with 20 μM HL142 for 36h and then with 6 ng/ml TGFβ at different time points, respectively. **C, D.** WB and densitometry analysis of p-FAK and total FAK expression in OVCAR3 and OVCAR8 cells treated with 20 μM HL142 for 36h and then 10 μM RGD at different time points. (***p<0.001, **p<0.01, *p<0.05).

Our previous studies also indicated that TGFβ induced EMT in OC cells 40, 41. Since HL142 inhibits EMT by decreasing the expression of ASAP1 and FAK in OC cells, we examined whether HL142 affects both the FAK and TGFβ pathways. We treated OVCAR3 and OVCAR8 cells with 10 μM HL142 for 6h and then treated cells with 6 ng/ml TGFβ at different time points. We found that HL142 significantly attenuated phospho-SMAD2 and the total SMAD2/3 in both OVCAR3 and OVCAR8 cells (Fig. 5A, B). We also examined the FAK pathway by treating OVCAR3 and OVCAR8 cells with 10 μM HL142 for 6h and then 10 µM integrin ligand cyclic RGD pentapeptide (Arg-Gly-Asp-D-Phe-Cys) to activate the FAK pathway at different time points. As shown in Fig. 5C and D, HL142 significantly attenuated the RGD-activated FAK pathway in both cell lines.

### HL142 inhibits primary ovarian tumor growth and metastasis in orthotopic OC mouse models

**Figure 6:**
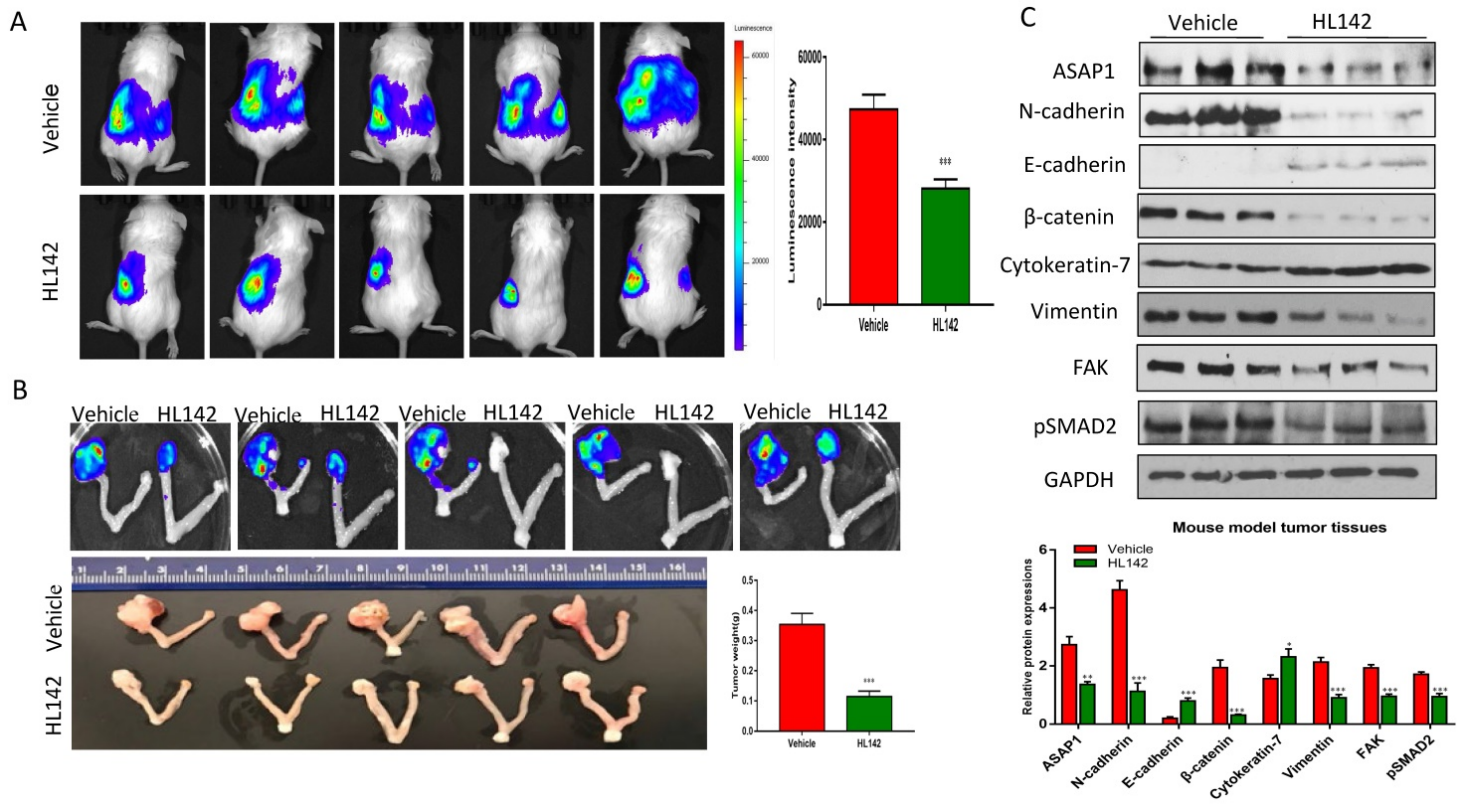
** HL142 inhibits primary ovarian tumor growth in orthotopic OC mouse models. A.** Bioluminescence images of ovarian tumors of mice at one month following intrabursal injection of OVCAR8-Luc2 cells and treated for every other day with HL142 (20 mg/kg body weight) or vehicle for three weeks (n=5).** B.** Primary ovarian tumors were imaged for bioluminescence, and tumors were measured and weighted following three weeks of treatment. **C**. WB and densitometry analysis of ASAP1, FAK, pSMAD2 and EMT markers in ovarian tumors from three different mice (***p<0.001, **p<0.01).

**Figure 7:**
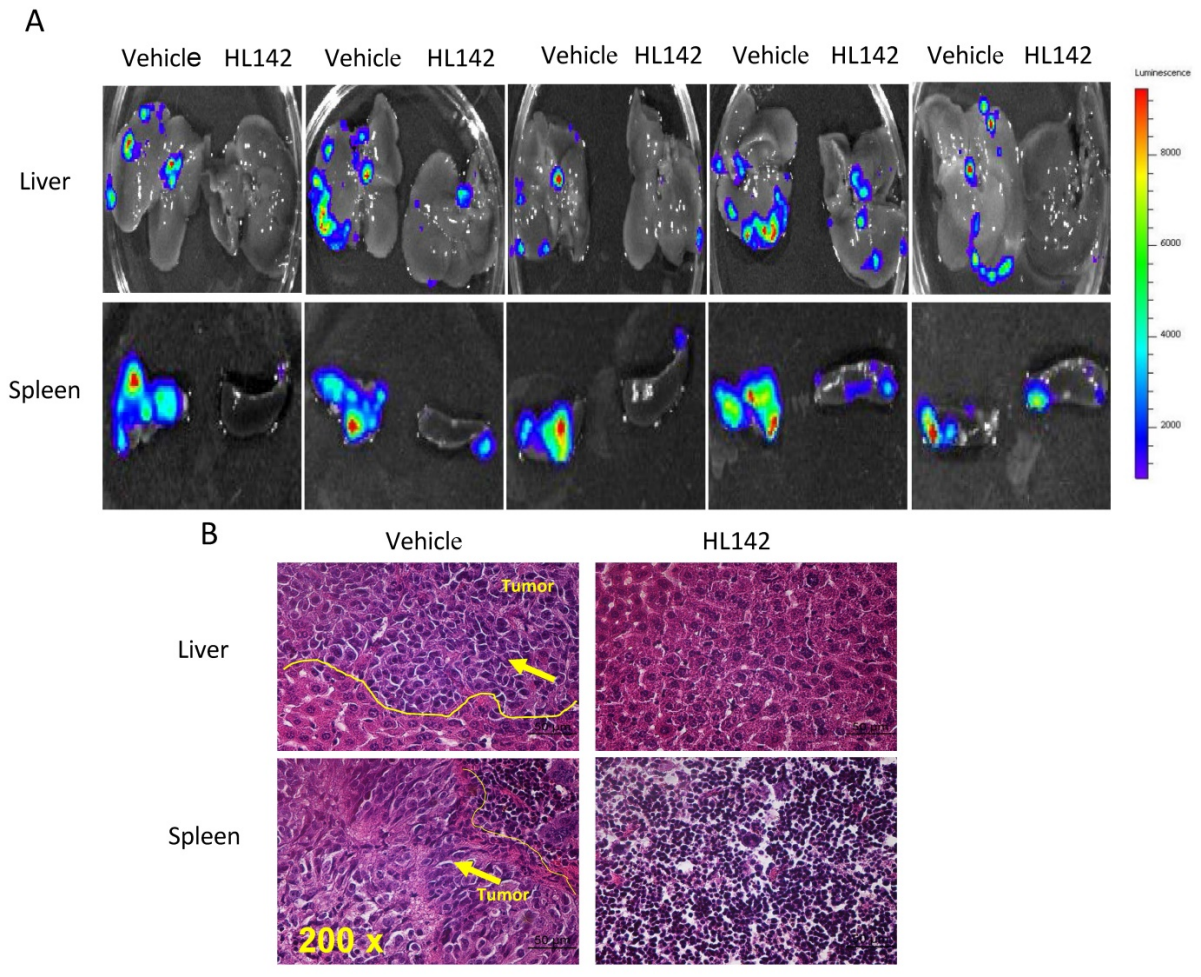
** HL142 inhibits ovarian tumor metastasis in an orthotopic OC mouse model. A.** Metastatic tumors in liver and spleen of xenografted mice were identified by bioluminescence imaging in five different mice treated with HL142 or vehicle. **B.** H&E stained metastatic tumors in liver and spleen of xenografted mice.

To test the efficacy of HL142 *in vivo*, we intrabursally injected wild-type OVCAR8-Luc2 cells into NSG female mice and treated them with HL142. We found that HL142 significantly inhibited primary OC growth and metastasis as shown by live animal imaging and comparing tumor weights between drug- and vehicle-treated mice (Fig. 6A, B). ASAP1, FAK, and EMT markers were also confirmed by Western blotting in primary ovarian tumors and their expression levels *in vivo* were consistent with the data obtained *in vitro*. APAP1, FAK, mesenchymal markers, and phospho-SMAD2 decreased significantly, whereas the epithelial markers E-cadherin and cytokeratin7 were upregulated in HL142-treated compared to vehicle-treated tumors (Fig. 6C). In addition, we detected tumor metastasis in these mice. As shown in Fig. 7A, metastatic tumors were observed in multiple peritoneal organs such as the liver and spleen of vehicle-treated mice but were barely found in organs of HL142-treated mice as shown by bioluminescent imaging. Tumors in livers and spleens from these mice were also confirmed by H&E staining (Fig. 7B). These results confirmed the above *in vitro* results that HL142 suppresses ovarian tumor growth and metastasis by inhibiting ASAP1, FAK, EMT and attenuating the FAK and TGFβ pathways.

## Discussion

OC rapidly becomes drug resistant and frequently recurs following initial chemotherapy. Identifying novel drug targets to reverse EMT can overcome drug resistance and improve cancer therapy by enhancing the efficacy of chemotherapy drugs through combination therapy. By screening a small compound library, we found that a synthetic analog of luminacin D, HL142, potently inhibits cell proliferation, migration, and invasion* in vitro*. Using an orthotopic OC mouse model, we demonstrated that HL142 effectively suppresses primary ovarian tumor growth and metastasis *in vivo*. Mechanistic studies suggested that HL142 inhibits ASAP1 and FAK, thus suppressing EMT by attenuating both FAK and TGFβ pathways in OC cells. We also found that HL142 sensitizes cell responses to chemotherapy drug treatment, indicating that HL142 is a potential drug in OC treatment.

ASAP1 contains a PH domain, a zinc finger, three ankyrin (ANK) repeats, a proline-rich region with SH3 binding motifs, eight repeats of the sequence E/DLPPKP, and an SH3 domain 42. Previous studies showed that ASAP1 directly interacts with the SH3 domains of tyrosine kinases including Src, Crk, p130^cas^, paxillin, and promotes cell growth 42. ASAP1 directly interacts with FAK via physical interaction between the C-terminal SH3 domain of ASAP1 with the second proline-rich motif in the C-terminal region of FAK 22. Interestingly, ASAP1 is upregulated and co-expressed with FAK in OC patients (Fig. 1A, B) and is associated with poor OC patient survival 29, while both ASAP1 and FAK are located in the same genomic locus 8q24, a well-known oncogenic locus. We showed here that ASAP1 indeed interacts with FAK in OC cells through immunoprecipitation (Fig. 1F). We found that HL142 inhibits both ASAP1 and FAK expression (Fig. 5, 6), while another luminacin D analog UCS15A disrupts the interaction between ASAP1 and its interaction proteins 36. Based on structural similarity between HL142 and UCS15A, we postulate that HL142 may function by directly inhibiting both ASAP1 and PTK2 expression in OC cells. The molecular mechanism underlying HL142 induced inhibition of ASAP1 and FAK is still not clear, which will be addressed in our future investigation in OC.

Our previous studies showed that ASAP1 promotes EMT in OC by activating EKR1/2 and APK pathways 29 and FAK has been shown to promote EMT in several cancer types 43-45. Since both ASAP1 and FAK promote EMT in OC cells and HL142 inhibits both ASAP1 and FAK expression, HL142 may reverse EMT by inhibiting ASAP1 and FAK in OC cells and also in orthotopic mouse models. Intraperitoneal delivery of chemotherapy drugs significantly improves OC therapy, and one of reasons is such drugs can directly target the mesenchymal-type tumor spheres to limit dissemination via inhibition of EMT. Our finding that HL142 inhibits EMT in OC cells provides a new avenue for therapy, which has been validated through *in vitro* cells and *in vivo* mouse models.

EMT is regulated by multiple signaling pathways in different cancers. In this study we showed that HL142 attenuates both FAK and TGFβ pathways. It is well known that activation of the FAK or TGFβ pathways promotes EMT in multiple cancer types 46-48. Therefore, it is not surprising to observe the attenuation of both pathways following HL142 treatment in OC cells. Although the luminacin analog UCS15A has been shown to inhibit tumor growth and metastasis, it is still unknown whether UCS15A inhibits EMT in OC. However, UCS15A has been shown to attenuate the Src kinase pathway by disrupting the interaction of proteins associated with Src, not by inhibiting Src activity 32. Src functions by forming a binary complex with FAK family kinases and, subsequently, phosphorylates other substrates and activates multiple signaling pathways 32, 49, 50. Previous studies showed that ASAP1 interacts with E-cadherin 51 and FAK cross-talks with the TGFβ pathway 52, 53. Therefore, it is possible that HL142 could regulate EMT by disrupting the interaction between ASAP1 and E-cadherin, which will require further validation in OC cells. We discovered that HL142, an analog of luminacin D, inhibits ovarian tumor growth and metastasis by suppressing EMT through attenuating the FAK and TGFβ pathways. Our finding provides the rationale and foundations to further evaluate and optimize the HL142 scaffold as a potential new approach for improved OC therapy in the future, either as a single agent or in combination with existing chemotherapy.

## Supplementary Material

Supplementary figures.
