# Supplementary material for: A Luminacin D Analog HL142 Inhibits Ovarian Tumor Growth and Metastasis by Reversing EMT and Attenuating the TGFβ and FAK Pathways
Source: J Cancer. 2021 Jul 25;12(18):5654–63. doi: 10.7150/jca.61066 (PMC8364639; doi:10.7150/jca.61066)
Supplement: Supplementary file 1 — Supplementary figures. [file jcav12p5654s1.pdf]

A luminacin D analog HL142 inhibits ovarian tumor growth and metastasis by reversing EMT and attenuating the TGFβ and FAK pathways

Baojin Wang, Hanxuan Li, Xinxin Zhao, Wenjing Zhang, Guannan Zhao, Zhongzhi Wu, Ruitao Zhang, Peixin Dong, Hidemichi Watari, Gabor Tigyi, Wei Li, Junming Yue

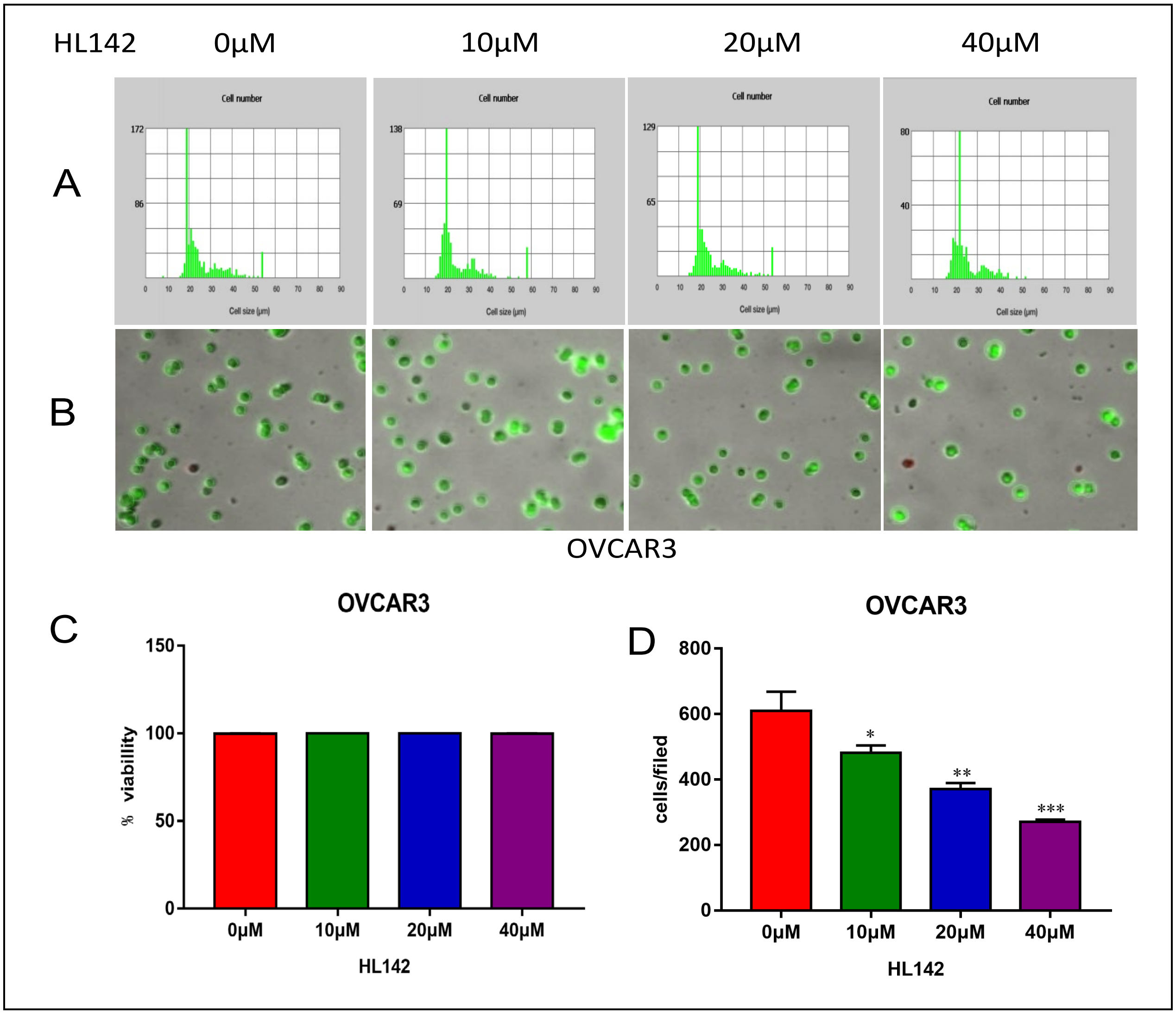

Fig. S1. Cell viability in OVCAR3 cells stained by AO/PI. A. Cell numbers relative to cell size in OVCAR3 cells treated with different doses of HL142 as shown by the Luna-FL automatic cell counter. **B**. Cell viability stained by AO/PI and live cells stained green and dead cells stained red. **C.** No significant differences were found on cell viability among different doses compared to vehicle treated cells. D. Cell proliferation measured by cell count (***p<0.001).

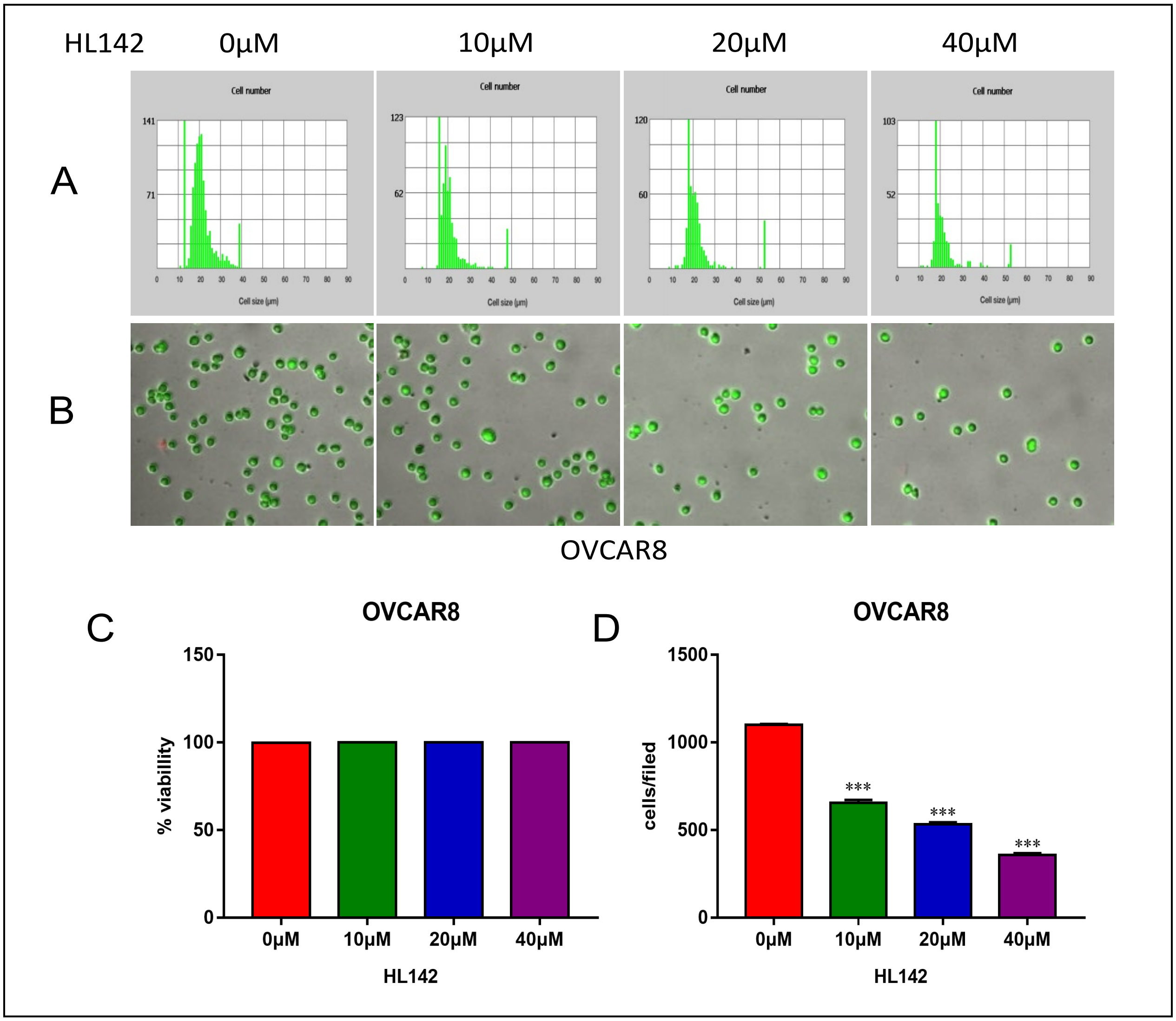

Fig.S2. Cell viability in OVCAR8 cells stained by AO/PI. **A.** Cell numbers relative to cell size in OVCAR8 cells treated with different doses of HL142 as shown by the Luna-FL automatic cell counter. **B**. Cell viability stained by AO/PI and live cells stained green and dead cells stained red. **C.** No significant differences were found on cell viability among different doses compared to vehicle treated cells. **D**. Cell proliferation measured by cell count (***p<0.001).
